# The relationship between digital technostress and cyber moral disengagement among college students: a moderated mediation model of psychological resilience, self-efficacy, and online self-control

**DOI:** 10.3389/fpsyg.2025.1706794

**Published:** 2025-11-11

**Authors:** Ruyan Hu, Qian Gao, Rufei Hu, Guofeng Liu, Liyuan Zhang

**Affiliations:** 1 School of Energy and Machinery, Dezhou University, Dezhou, China; 2 School of Education Science, Yangzhou University, Yangzhou, China; 3 The Affiliated Taizhou People’s Hospital of Nanjing Medical University, Taizhou, China

**Keywords:** digital technostress, cyber moral disengagement, psychological resilience, self-efficacy, online self-control

## Abstract

This study explores the relationship between digital technostress and cyber moral disengagement among college students, with a particular focus on the mediating role of psychological resilience, self-efficacy, and the moderating role of online self-control. This study conducted a questionnaire survey on 1980 college students using the Digital Technostress Scale, Cyber Moral Disengagement Questionnaire, Internet Usage Self- Control Scale, Psychological Resilience Scale, and Self-efficacy Scale. The results indicate that: (1) There is a significant positive correlation between digital technostress and cyber moral disengagement; (2) The independent and chain mediated pathways of psychological resilience and self-efficacy between digital technostress and cyber moral disengagement are established; (3) The online self-control plays a moderating role in the relationship between digital technostress and cyber moral disengagement. In summary, this study highlights the mediating role of psychological resilience and self-efficacy, as well as the moderating role of online self-control. This study advances theoretical understanding of the mechanisms linking digital technostress and cyber moral disengagement, and underscores the pivotal role of online self-control in mitigating cyber moral disengagement among college students.

## Introduction

With the rapid and iterative advancement of generative artificial intelligence technologies, such as Kimi, ChatGPT, and DeepSeek, Internet use has become deeply integrated into individuals’ daily learning, work, and life. As of February 2025, the global number of Internet users was estimated at 5.56 billion (Statista, 2025). As of June 2024, the number of Internet users in China had reached nearly 1.1 billion, with adolescents aged 10 to 19 accounting for 49% of newly added users (CNNIC, 2024). College students have emerged as one of the most active subgroups of “digital natives” engaging in Internet use across multiple domains, including social interaction (Lee et al., 2023), information acquisition (Salari et al., 2025), knowledge learning (Pham Thi and Duong, 2024), and entertainment (Romero-López et al., 2021). As an extension of traditional morality into the online sphere, robust online moral literacy promotes college students’ compliance with Internet regulations, rational expression of opinions, and protection of personal privacy (Lee and Jun, 2024), while cultivating a strong sense of responsibility and moral judgment in virtual environments. However, in practice, some college students engage in behaviors indicative of cyber moral disengagement, including the misappropriation of online content (Pavlovic et al., 2024; Fajt and Schiller, 2025), rationalization of data falsification (Dias-Oliveira et al., 2024), verbal aggression, and privacy violations. In addition, variations in online moral literacy have been observed across gender, academic year, and frequency of Internet use. Specifically, female students tend to outperform male students in online moral cognition and behavioral restraint, and those in higher academic years generally demonstrate stronger online moral restraint. Conversely, individuals with excessively high Internet use frequency face an elevated risk of cyber moral disengagement, as their online moral judgment is more vulnerable to impulsivity and conformity pressures (Mardianto et al., 2021).

In this context, the issue of cyber moral disengagement among university students and its influencing factors (e.g., psychological traits, social norms) has garnered growing scholarly attention, with situational variables-particularly digital technostress-being identified as significant contributors (Lv, 2022; Fissel et al., 2025; Li et al., 2025). Digital technostress, as an emerging source of psychological strain, permeates multiple domains-including higher education, peer interaction, and platform usage-and may trigger diverse online problems via mechanisms such as heightened cognitive load and impaired emotional regulation (Angioletti and Fronda, 2024). Moreover, a growing body of research has begun to explore the psychological mechanisms linking these influencing factors to cyber moral disengagement.

The Theory of Planned Behavior (TPB; Ajzen, 1991) is a prominent psychological framework frequently employed to predict and explain individual behavioral intentions. According to TPB, intentions are determined by three fundamental components: attitudes toward the behavior (i.e., evaluations of expected outcomes), subjective norms (i.e., perceived social expectations), and perceived behavioral control (i.e., one’s perceived capacity to exert control over the behavior). Specifically, belief-related factors (e.g., perceived usefulness, perceived risk) are considered key determinants of attitudes toward behavior, whereas outcome-evaluation factors (e.g., value weighting) may hinder the formation and enactment of behavioral intentions (Sussman and Gifford, 2019). Belief and evaluative factors jointly are associated with individuals’ perceptions of social norms and their sense of behavioral control, which in turn may play a role in the propensity to engage in morally disengaged behaviors. Beyond explaining individual behavioral intentions, TPB has been widely applied to the study of group-level attributes and online literacy. Notably, among the subjective norm factors encompassed by TPB, digital technostress emerges as a critical disruptive variable that warrants further empirical exploration.

Previous research suggests that individuals experiencing high levels of digital technostress often report reduced perceived self-control and are prone to heightened emotional load, attentional distraction, and cognitive biases in real-world contexts (Khetawat and Steele, 2023). To alleviate such cognitive-emotional discomfort, these individuals may rely on virtual environments as a means of escape or compensation. When heightened stress coincides with reduced perceived control, individuals are more prone to maladaptive online behaviors, including verbal aggression, privacy breaches, and information misuse (Grande et al., 2020). In addition, previous research has highlighted a significant link between perceived social pressure and the development of individual behavioral intentions (Huyen et al., 2022). However, research has yet to investigate in depth the mechanisms through which digital technostress is associated with cyber moral disengagement among university students. Drawing on TPB, this study posits that psychological resilience-defined as the capacity to adapt effectively to adversity-and self-efficacy-referring to individuals’ confidence in their ability to perform specific tasks-serve as mediating mechanisms linking digital technostress to cyber moral disengagement.

Existing research on cyber moral disengagement has paid limited attention to protective factors and their interaction with potential risk factors (Ma et al., 2024). Related studies have identified online self-control as a protective factor against cyber moral disengagement (Kim and Lee, 2021). Online self-control operates through self-restraint and delayed gratification, thereby mitigating the impact of digital technostress on cyber moral disengagement (Su et al., 2023). This study seeks to examine both the independent and sequential mediating relationships of psychological resilience and self-efficacy, along with the moderating relationship of online self-control, in the association between digital technostress and cyber moral disengagement.

### Digital technostress and cyber moral disengagement

Digital technostress (DTS) is a situational psychological stressor, manifested as tension or fatigue resulting from prolonged exposure to information overload or complex digital environments, often accompanied by anxiety, diminished attention, and value conflicts (Balconi et al., 2017; Crivelli and Balconi, 2017). Cognitive Load Theory (CLT) emphasizes that when individuals are confronted with information stimuli exceeding their cognitive capacity (Sweller, 1988), cognitive resources become overtaxed and working memory overloaded, thereby impairing problem-solving and value judgment abilities. In digital contexts, individuals subjected to sustained high cognitive load not only experience heightened psychological fatigue but also exhibit impaired comprehensive assessment of online behavioral consequences, making them more prone to immediate, impulsive reactions rather than value-guided deliberation. This state often gives rise to what is termed “technologically induced learned helplessness”. Digital technostress represents a deep-seated cognitive interference factor that erodes individuals’ intrinsic adherence to institutional norms and is associated with lower self-efficacy, a diminished sense of value, and a blurred sense of responsibility in online environments. TPB emphasizes the multifactorial interaction of behaviors, applicable to specific beliefs and outcome evaluations, whereas CLT focuses on automated, irrational responses arising from cognitive overload in specific contexts (i.e., high load) (Minkley et al., 2021). This framework is also applicable to cyber moral disengagement. Online disinhibition relationships (e.g., anonymity) (Varghese, 2025) further amplify the erosive relationship of digital technostress on moral regulatory mechanisms. When low perceived behavioral consequences (e.g., anonymity shielding speech from accountability) are coupled with high emotional compensation needs (e.g., immediate gratification from venting), digital technostress may be more likely to translate into cyber moral disengagement behaviors. Additionally, a positive moderating relationship of digital technostress on moral contextual judgment (Barque-Duran et al., 2017). Therefore, this study posits hypothesis 1: Digital technostress is significantly positively associated with cyber moral disengagement.

### The mediating mechanisms of psychological resilience and self-efficacy

Psychological resilience is defined as the capacity of individuals to adapt effectively, recover, and even achieve positive growth in the face of trauma or major life events (Nishimi et al., 2021). In a society where digitalization is deeply embedded, prolonged exposure to digital technostress can impose sustained psychological burdens and cognitive exhaustion, ultimately diminishing individuals’ psychological resilience. Problem-solving ability constitutes a core component of psychological resilience, and prior research has demonstrated that this capability often remains effective even under stressful conditions (Largo-Wight et al., 2005). Moreover, research suggests that individuals with higher psychological resilience are better able to actively manage online conflicts and stress, preserve moral sensitivity and adherence to behavioral norms, and avert passive or maladaptive responses (Gefen et al., 2025). According to TPB, perceived behavioral control is associated with both behavioral intentions and actual behaviors, which may is linked to behavioral deviations in specific contexts. Individuals exposed to prolonged digital technostress are more likely to display maladaptive psychological responses-such as cognitive avoidance, emotional breakdown, or value dissonance-during problem-solving processes. Such negative cognition, for example, perceiving online technology as an uncontrollable threat or positioning oneself as a passive victim-are associated with lower mood (Ali et al., 2022), reduced responsibility-taking, and even moral numbness. Consequently, individuals with low psychological resilience are more likely to rely on coping strategies such as avoidance or denial, which in turn reduces their sensitivity to social norms. The anonymity afforded by online environments further attenuates external normative pressures, making those with lower resilience more prone to disregard societal expectations (Chou et al., 2017). Additionally, research indicates a significant association between mental health, digital technostress, and online behavior (Li et al., 2025). Based on these findings, this study proposes Hypothesis 2: Psychological resilience mediates the relationship between digital technostress and cyber moral disengagement.

Self-efficacy, defined as an individual’s assessment of their ability to successfully perform specific tasks (Bandura, 1977), may act as a key mechanism connecting digital technostress to cyber moral disengagement. Individuals experiencing digital technostress often exhibit diminished perceived control, arising from contextual factors such as cognitive overload, frequent system changes, and attentional distractions. Furthermore, pressures associated with digital transformation may undermine confidence in task execution, thereby reducing digital self-efficacy (Zhao, 2025). Within the framework of TPB, perceived behavioral control may play a role in the execution of behavioral intentions, which subsequently shape individuals’ perceptions of subjective norms. As a key manifestation of perceived behavioral control, self-efficacy fosters proactive anticipation of moral challenges (e.g., “I can resist temptation”) and strengthens the perceived threat of behavioral consequences (Elemo and Temtime, n.d.), thereby constraining the translation of intentions for cyber moral disengagement into actual behaviors. Previous studies have indicated that low self-efficacy depletes self-regulatory resources, leaving individuals with insufficient internal control when confronted with online temptations or conflicts, thereby heightening the likelihood of aggressive language or behaviors (Favini et al., 2024). Building on the theoretical rationale, this study proposes a series of hypotheses regarding the mechanisms linking digital technostress to cyber moral disengagement. Specifically, it is hypothesized that self-efficacy mediates the association between digital technostress and cyber moral disengagement (Hypothesis 3). Furthermore, psychological resilience and self-efficacy are expected to function as sequential mediators, jointly explaining the process through which digital technostress is associated with cyber moral disengagement (Hypothesis 4).

We hypothesize a sequential mediation pathway where psychological resilience precedes self-efficacy. This proposed ordering is grounded in the conceptual distinction between a general disposition and a specific, malleable belief. We define resilience as a broad, trait-like resource that determines one’s foundational capacity to adapt to adversity. In contrast, self-efficacy is a more context-specific belief in one’s ability to execute a task. We theorize that when confronted by technostress, an individual’s general resilience first acts as a buffer. This adaptive response, in turn, preserves or fosters their specific self-efficacy for managing online challenges. This sequence, from a general resource to a specific belief, offers a more nuanced and theoretically coherent explanation of the psychological processes linking technostress to moral cognitions.

### The moderating role of online self-control

Although digital technostress may indirectly is associated with cyber moral disengagement through mediating variables such as psychological resilience and self-efficacy, the strength and direction of these relationships are likely to vary across individuals. Online self-control defined as an individual’s capacity to regulate behavior in the face of Internet temptations, primarily through strategies such as self-restraint and delayed gratification (Tangney et al., 2004) has been shown to mitigate cyber moral disengagement. Individuals with higher levels of online self-control are better able to maintain goal-directed behavior, monitor their online actions, promptly correct potential moral lapses, and sustain consistent value-driven responses (Shaigerova et al., 2021). Based on these insights, this study further proposes Hypothesis 5: Online self-control moderates the indirect relationships of digital technostress on cyber moral disengagement via psychological resilience and self-efficacy, such that the mediating relationships are stronger for individuals with higher levels of online self-control.

In online contexts, individuals experiencing digital technostress tend to exhibit cognitive appraisals and behavioral decision-making patterns that are associated with their level of online self-control, which serves as a key psychological regulatory mechanism. Specifically, individuals with strong self-control typically perceive cyber moral disengagement as deviant behavior (Whitten et al., 2024) and demonstrate robust executive control functions, such as the inhibition of immediate impulses (Baumeister et al., 2007). They are less susceptible to cognitive distortions (e.g., “occasional lapses are harmless”) and can effectively prevent stress from translating into disengaged behavior, thereby buffering against moral disengagement driven by cognitive overload or emotional exhaustion. Conversely, individuals with low self-control are more susceptible to the cognitive bias of ‘impulse rationalization,’ in which they amplify situational pressures (e.g., “the situation compelled me to act”) to justify morally deviant behavior. This cognitive mechanism enables individuals to engage in cyber moral disengagement without experiencing guilt or psychological distress.

Related research suggests that trait self-control can mitigate the harmful relationships of deviant peer associations among morally vulnerable individuals (Hirtenlehner et al., 2022). Prior studies have also confirmed that deficits in self-regulatory capacity are a core characteristic of individuals engaging in moral disengagement, and that the cultivation of self-regulation can prevent both rule-breaking and moral disengagement (Bembenutty, 2023). In addition, scholars have emphasized that certain cognitive regulatory factors (e.g., metacognitive monitoring) may be associated with the pathways through which psychological factors influence individuals’ online behavioral performance, particularly by enhancing self-awareness and goal-directed action that help mitigate the adverse effects of external stress (Smeets and Quaedflieg, 2019). Collectively, these findings offer indirect theoretical evidence for the function of online self-control as a psychological regulatory mechanism. Accordingly, this study proposes Hypothesis 5: Online self-control moderates the relationships among digital technostress, psychological resilience, self-efficacy, and cyber moral disengagement.

### The current study

**Figure 1:**
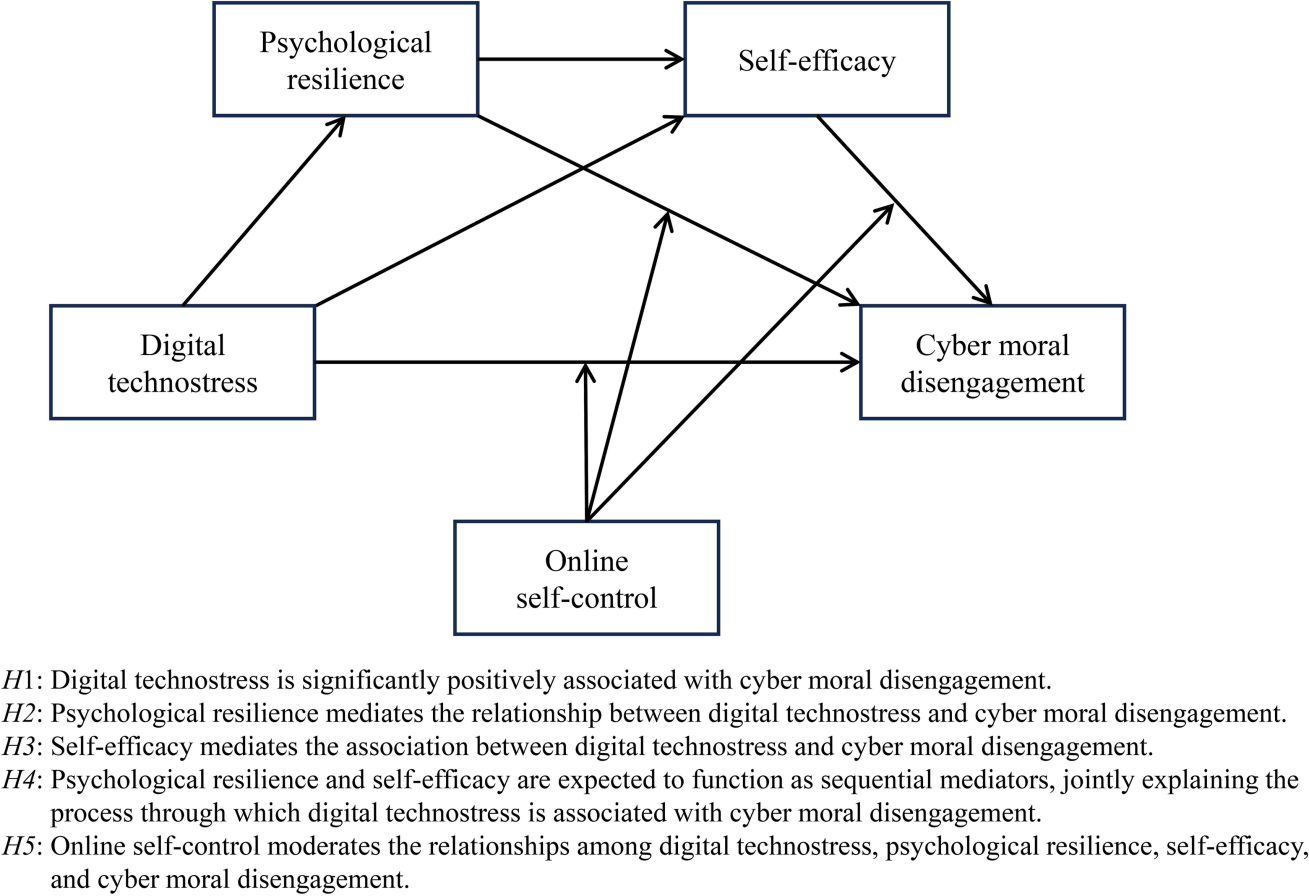
Conceptual hypothesis model.

The present study recruited university students in China as its research sample. Data indicate that 87.45% of participants perceive the Internet as a medium for learning, social interaction (e.g., Weibo, WeChat), and entertainment (e.g., Douyin, Kuaishou), with 28.54% reporting daily Internet usage exceeding 6 h. As typical representatives of “digital natives” (Prensky, 2001), university students generally exhibit higher levels of self-awareness and social responsibility than adolescents, rendering them more likely to participate in public online discussions on diverse issues. Moreover, Xiao et al. (2022) reported that older adolescents display greater behavioral regulation and lower levels of cyber moral disengagement when confronted with online moral dilemmas, relative to their younger counterparts. Existing research has predominantly concentrated on adolescent populations, with relatively few studies examining university students. Therefore, the present study focuses on university students to empirically test the aforementioned hypotheses. The conceptual model of the study is illustrated in Figure 1.

## Methods

### Participants

A total of 2,109 undergraduate students from five universities across Shandong, Jiangsu, Henan, Hebei, and Inner Mongolia were recruited for participation in this study (The detailed information can be found in the Supporting Materials, Section I: Demographic Profile). Following a thorough screening procedure to eliminate invalid responses, 1,980 valid questionnaires were retained (1,010 males and 970 females). The sample comprised 43.69% first-year students (*n* = 865), 28.54% second-year students (*n* = 565), 21.46% third-year students (*n* = 425), and 6.31% fourth-year students (*n* = 125).

Regarding family structure, 32.83% of participants came from one-child families, whereas 67.17% were from multi-child families. Regarding academic majors, participants represented diverse disciplines, including humanities, natural sciences, engineering, medicine, arts, and physical education; the majority (65.15%) were enrolled in science and engineering programs. With respect to place of origin, 66.16% of participants came from rural areas, while 33.84% were from urban areas. Additionally, data on daily Internet usage were collected, indicating that 67.68% of students reported using the Internet for more than 3 h per day. All items were rated on 5-point Likert scales and were treated as continuous variables. Statistical analyses were conducted using IBM SPSS Statistics (version 26.0). Mediation and moderated mediation effects were estimated using the PROCESS macro (Model 6 and Model 15) based on ordinary least squares regression with 5,000 bootstrap samples. The estimation followed the maximum likelihood assumption for continuous observed indicators.

## Measures

### Digital technostress measurement

The Chinese version of the College Student Digital Technostress Scale was employed to assess digital technostress among college students (Chen, 2025, the detailed information can be found in the Supporting Materials, Section II: Digital technostress). Previous research has shown that this scale possesses satisfactory reliability and validity. The Digital Technostress Scale consists of 47 items across 11 dimensions (e.g., technostress overload, techno-invasion, techno-complexity, etc.). Each item was rated on a five-point Likert scale from 1 (strongly disagree) to 5 (strongly agree). Higher scores indicate greater levels of perceived digital technostress. To validate the hierarchical structure of this scale, a higher-order CFA was conducted using AMOS 28.0. This model specified the 11 distinct first-order factors (dimensions) to load onto a single second-order latent factor representing overall Digital Technostress. The fit indices for this higher-order model demonstrated acceptable fit: *χ*^2^/df = 2.461, CFI = 0.930, TLI = 0.920, RMSEA = 0.061, and SRMR = 0.078. The overall Cronbach’s *α* and McDonald’s *ω* for the scale were 0.961 and 0.962, respectively. Standardized factor loadings for all items on their respective first-order factors ranged from 0.62 to 0.88, and the first-order factors showed good loadings on the second-order factor, ranging from 0.71 to 0.91. The composite reliability (CR) for the second-order factor was 0.94, and the average variance extracted (AVE) was 0.68, indicating good reliability and convergent validity for the overall construct.

### Cyber moral disengagement questionnaire

Cyber moral disengagement among Chinese college students was assessed using the Cyber Moral Disengagement Questionnaire, adapted by Zhang (2015) from the Adolescent Online Deviant Behavior Scale originally developed by Lei (2008). This instrument has been widely applied in research involving Chinese college populations (Zhang and Yutong, 2025, the detailed information can be found in the Supporting Materials, Section III: Cyber moral disengagement). The questionnaire comprises four dimensions: online interaction deviation (e.g., “indulging in making friends with strangers online”), online verbal deviation (e.g., “verbally attacking others online”), online pornography (e.g., “browsing pornographic websites”), and online usage deviation (e.g., “posting or forwarding false statements online”). The instrument includes 19 items, each rated on a five-point Likert scale ranging from 1 (strongly disagree) to 5 (strongly agree), with higher scores indicating greater engagement in cyber moral disengagement behaviors. Confirmatory factor analysis (CFA) conducted in this study yielded fit indices of *χ*^2^/df = 2.972, CFI = 0.976, TLI = 0.964, RMSEA = 0.071, and SRMR = 0.015. The Cronbach’s *α* coefficients for online interaction, online verbal, online pornography, online usage, and the total scale were 0.900, 0.951, 0.927, 0.957, and 0.973, and the McDonald’s *ω* coefficients for online interaction, online verbal, online pornography, online usage, and the total scale were 0.904, 0.952, 0.930, 0.957, and 0.975, indicating excellent internal consistency across all dimensions.

### Online self-control scale

Online self-control among Chinese college students was assessed using the Chinese version of the Internet Usage Self-Control Scale for College Students (IUSCS-CS), developed by Ouyang et al. (2013). This scale has been widely applied in research involving Chinese college student populations (Li et al., 2018, the detailed information can be found in the Supporting Materials, Section II: Online self-control). It comprises three dimensions: cognitive (e.g., “the Internet has a negative impact on my life”), emotional (e.g., “I become irritated when disturbed while online”), and behavioral self-control (e.g., “I often act impulsively online”). The scale includes 34 items, each rated on a five-point Likert scale from 1 (strongly disagree) to 5 (strongly agree), with higher scores indicating greater levels of online self-control. Confirmatory factor analysis (CFA) in the present study demonstrated acceptable model fit, with *χ*^2^/df = 2.700, CFI = 0.920, TLI = 0.897, RMSEA = 0.066, and SRMR = 0.020. The Cronbach’s *α* coefficients for the cognitive, emotional, behavioral, and total scale were 0.791, 0.703, 0.770, and 0.884, and the McDonald’s ω coefficients for the cognitive, emotional, behavioral, and total scales were 0.797 0.716, 0.808, and 0.917, indicating acceptable to good internal consistency across all dimensions.

### Psychological resilience scale

Based on Sinclair and Wallston (2004) Brief Resilient Coping Scale, Yang (2005) developed the Resilience Scale for University Students (RSUS) to assess psychological resilience among Chinese college students. This instrument has demonstrated good reliability and validity in prior research (Wu and Huang, 2024, the detailed information can be found in the Supporting Materials, Section V: Psychological resilience). The scale comprises six dimensions: self-acceptance (e.g., “I think I am still a good person”), self-efficacy (e.g., “I am confident in my abilities”), emotional stability (e.g., “I often feel down for no reason”), problem-solving (e.g., “I am good at allocating my time effectively to solve problems”), peer support (e.g., “I have at least one friend with whom I can share everything”), and family support (e.g., “When I face difficulties, I often receive substantial support from my family”). The RSUS consists of 31 items, each rated on a five-point Likert scale from 1 (strongly disagree) to 5 (strongly agree), with higher scores reflecting greater levels of psychological resilience. Confirmatory factor analysis (CFA) conducted in this study yielded fit indices of *χ*^2^/df = 2.770, CFI = 0.941, TLI = 0.924, RMSEA = 0.067, and SRMR = 0.051. Cronbach’s *α* coefficients for self-acceptance, self-efficacy, stability, problem-solving, peer support, family support, and the total scale were 0.873, 0.791, 0.761, 0.878, 0.882, 0.924, and 0.950, and the McDonald’s *ω* coefficients for self-acceptance, self-efficacy, stability, problem-solving, peer support, family support, and the total scale were 0.875, 0.829, 0.786, 0.879, 0.902, 0.926, and 0.960, indicating good to excellent internal consistency across all dimensions.

### Self-efficacy scale

Self-efficacy among Chinese college students was assessed using the Chinese version of the General Self-Efficacy Scale (GSES), originally developed by Schwarzer et al. and translated and adapted by Wang et al. (2001) (the detailed information can be found in the Supporting Materials, Section VI: self-efficacy). The GSES consists of 10 unidimensional items, such as “If I try hard enough, I can always solve the problem,” each rated on a four-point Likert scale ranging from 1 (not at all true) to 4 (exactly true), with higher scores indicating greater levels of self-efficacy. Confirmatory factor analysis (CFA) in the present study demonstrated good model fit (*χ*^2^/df = 2.747, CFI = 0.988, TLI = 0.977, RMSEA = 0.067, SRMR = 0.020), and the scale showed excellent internal consistency with Cronbach’s α of 0.947 and McDonald’s ω of 0.948.

### Data analyses

All constructs were measured using multi-item Likert scales (primarily 5-point). In line with common practice in psychological and social science research, composite scores (means) were calculated for each construct. These composite scores were treated as continuous variables for all subsequent analyses. This approach is considered robust when scales consist of multiple items and have five or more response categories, as the resulting composite variables approximate continuous measurement. The primary hypotheses were tested using the PROCESS 4.1 macro for SPSS 26.0. This tool employs an Ordinary Least Squares (OLS) regression-based path analysis approach to estimate direct, indirect, and conditional effects. To address the potential non-normality of the sampling distribution of indirect effects, we utilized a non-parametric bootstrapping procedure. Bias-corrected 95% confidence intervals were generated from 5,000 bootstrap resamples. An effect was considered statistically significant if its confidence interval did not include zero. Prior to analysis, all continuous predictor and moderator variables were mean-centered to reduce multicollinearity and facilitate the interpretation of interaction terms. Preliminary correlational analyses were conducted in SPSS using the default Maximum Likelihood (ML) estimator.

CFA was first conducted using AMOS 28.0 to validate the measurement models and confirm the reliability and discriminant validity of each construct. With model fit evaluated through indices including *χ*^2^/df, RMSEA, CFI, TLI, and SRMR. All scales in this study met commonly accepted thresholds *χ*^2^/df < 5, RMSEA < 0.08, CFI > 0.90, TLI > 0.80, SRMR < 0.08 (Chen, 2007), indicating acceptable to good model fit. Because AMOS does not directly compute bias-corrected bootstrapped confidence intervals for complex sequential mediation models, factor scores derived from the validated CFAs were exported into SPSS 26.0. The mediating relationships of psychological resilience and self-efficacy were then performed using Hayes’ PROCESS 4.1 macro (Bolin, 2014), (Model 6) with 5,000 bootstrap samples. This hybrid approach preserved the benefits of latent variable validation while allowing robust estimation of indirect and sequential mediation effects, ensuring methodological consistency and statistical rigor. Three specific mediation pathways were examined: (1) the mediating relationship of self-efficacy, (2) the mediating relationship of psychological resilience, and (3) the sequential mediating relationship of psychological resilience and self-efficacy. For each pathway, 95% confidence intervals (95% CIs) were calculated; intervals containing zero indicated non-significant relationships, whereas intervals not containing zero indicated statistically significant mediation (Shrout and Bolger, 2002).

Finally, the moderating relationship of online self-control was examined using Model 15 of the PROCESS 4.1 macro, with 5,000 bootstrap samples. The significance of moderation was determined by examining the relationships of the interaction terms between digital technostress and online self-control, as well as between psychological resilience and online self-control, on cyber moral disengagement. To further investigate the moderating role of online self-control, the Johnson-Neyman (J-N) technique (Johnson and Fay, 1950) was applied to conduct simple slope analyses and to determine the transition point at which online self-control significantly moderates the relationship. When the online self-control score was above or below the transition point, the significance of the moderating relationship on the relationships between digital technostress and cyber moral disengagement, and between psychological resilience and cyber moral disengagement, was determined based on whether the 95% *CI* of the conditional relationship included zero.

## Results

### Common-method bias test and collinearity diagnosis

As all data in this study were collected via a questionnaire survey, there exists a potential for common method bias (CMB). To assess the potential impact of CMB, Harman’s single-factor test was performed using unrotated principal component factor analysis across all measured variables (Harman, 1976). The analysis revealed 20 factors with eigenvalues greater than 1, with the first factor accounting for 22.85% of the total variance, which is below the critical threshold of 40% (Podsakoff et al., 2003). These findings suggest that the study is not substantially associated with by common method bias. Furthermore, to examine potential multicollinearity, variance inflation factor (VIF) diagnostics were conducted, revealing that all variables had VIF values ranging from 1.99 to 8.68 (all < 10), indicating the absence of substantial multicollinearity.

### Descriptive statistics and correlation analysis

To preliminarily explore the relationships among the study variables, descriptive statistics and Pearson correlation analyses were conducted. The means, standard deviations, and correlation coefficients for online self-control, cyber moral disengagement, digital technostress, self-efficacy, and psychological resilience are presented in Table 1. Significant correlations were observed across all variables. Notably, self-efficacy and psychological resilience (*r* = 0.241, *p* < 0.01), digital technostress and psychological resilience (*r* = 0.478, *p* < 0.01), and online self-control and psychological resilience (*r* = 0.258, *p* < 0.01) were positively correlated, whereas the remaining variable pairs exhibited significant negative correlations.

**Table 1:** Descriptive statistics and correlation matrix of study variables.

Variables	1	2	3	4	5	6	7	8	9
1. Gender	1.000								
2. Grade	0.237^**^	1.000							
3. Only child or not	0.099^*^	−0.030	1.000						
4. Daily internet usage time	0.101^*^	−0.026	0.101^*^	1.000					
5. Online self-control	−0.056	−0.085	0.000	0.137^**^	1.000				
6. Cyber moral disengagement	−0.157^**^	−0.199^**^	−0.101^*^	0.039	0.392^**^	1.000			
7. Digital technostress	0.092	−0.023	0.032	0.087	0.452^**^	0.171^**^	1.000		
8. Self-efficacy	−0.062	−0.023	−0.015	−0.052	−0.157^**^	−0.267^**^	−0.220^**^	1.000	
9. Psychological resilience	0.100^*^	0.089	−0.012	−0.011	0.258^**^	−0.207^**^	0.478^**^	0.241^**^	1.000
*M*					2.319	1.405	2.909	2.660	3.462
SD					0.493	0.630	0.642	0.588	0.676

*N* = 1980. M, mean; SD, standardized deviation. *p<0.05; **p<0.01.

### Multiple mediation analysis of psychological resilience and self-efficacy between digital technostress and cyber moral disengagement

All variables were standardized prior to analysis. Controlling for gender, academic year, only-child status, and daily internet use duration, a sequential mediation model was tested, with digital technostress as the independent variable, cyber moral disengagement as the dependent variable, and psychological resilience and self-efficacy as mediators. Mediation relationships were examined using Model 6 of the PROCESS macro with 5,000 bootstrap samples. The model exhibited acceptable fit indices (*χ*^2^/df = 2.338, RMSEA = 0.058, CFI = 0.955, TLI = 0.946, SRMR = 0.045; see Table 2).

**Table 2:** Mediation analysis of psychological resilience and self-efficacy.

Variable	Model 1 (Cyber moral disengagement)			Model 2 (Self-efficacy)			Model 3 (Psychological resilience)			Model 4 (Cyber moral disengagement)		
	*β*	SE	*t*	*β*	SE	*t*	*β*	SE	*t*	*β*	SE	*t*
Constant	0.999	0.254	3.935^***^	0.231	0.260	0.887	−0.231	0.213	−1.085	0.992	0.239	4.149^***^
Gender	−0.299	0.118	−2.534^*^	−0.081	0.121	−0.665	0.124	0.099	1.252	−0.285	0.111	−2.558^*^
Grade	−0.205	0.061	−3.355^***^	−0.025	0.063	−0.403	0.119	0.051	2.323^*^	−0.177	0.058	−3.075^**^
Only child or not	−0.221	0.104	−2.134^*^	−0.004	0.106	−0.037	−0.053	0.087	−0.616	−0.237	0.097	−2.437^*^
Daily internet usage time	0.037	0.043	0.872	−0.027	0.044	−0.615	−0.160	0.036	−0.449	0.026	0.040	0.646
Digital technostress	0.179	0.049	3.647^***^	−0.214	0.050	−4.307^***^	0.559	0.042	13.452^***^	0.283	0.057	5.005^***^
Self-efficacy							0.368	0.041	8.893^***^	−0.148	0.051	−2.903^**^
Psychological resilience										−0.283	0.057	−4.968^***^
*R^2^*	0.096			0.051			0.370			0.207		
*F*	8.277^***^			4.217^***^			38.029^***^			14.431^***^		

*N* = 1980. *p<0.05; **p<0.01; ***p<0.001.

In Model 1, digital technostress was significantly positively associated with cyber moral disengagement (*β* = 0.179, *SE* = 0.049, *t* = 3.647, *p* < 0.001), supporting Hypothesis 1. In Model 2, digital technostress was significantly negatively associated with self-efficacy (*β* = −0.214, *SE* = 0.050, *t* = −4.307, *p* < 0.001). In Model 4, self-efficacy was significantly negatively associated with cyber moral disengagement (*β* = −0.148, *SE* = 0.051, *t* = −2.903, *p* < 0.001), indicating that the mediation path “digital technostress → self-efficacy → cyber moral disengagement” was supported, thereby confirming Hypothesis 3.

In Model 3, digital technostress was significantly and positively associated with psychological resilience (*β* = 0.559, *SE* = 0.042, *t* = 13.452, *p* < 0.001). In Model 4, psychological resilience was significantly and negatively related to cyber moral disengagement (*β* = −0.283, *SE* = 0.057, *t* = −4.968, *p* < 0.001), supporting the mediation pathway ‘digital technostress → psychological resilience → cyber moral disengagement’ and thereby confirming Hypothesis 2.

Finally, in Model 3, psychological resilience was significantly and positively associated with self-efficacy (*β* = 0.368, *SE* = 0.041, *t* = 8.893, *p* < 0.001). Combined with the previously established paths “digital technostress → psychological resilience” and “self-efficacy → cyber moral disengagement,” the sequential mediation path “digital technostress → psychological resilience → self-efficacy → cyber moral disengagement” was supported, confirming Hypothesis 4.

To further assess the significance of the mediation relationships, a bias-corrected bootstrap procedure with 5,000 resamples was conducted, with results presented in Table 3. The total effect was 0.179 [SE = 0.049, 95% CI = (0.083, 0.274)], direct effect was 0.283 [SE = 0.057, 95% CI = (0.172, 0.394)], indirect effect was −0.104 [SE = 0.043, 95% CI = (−0.191, −0.024)]; as the confidence interval did not include zero, the relationship was statistically significant. Both psychological resilience and self-efficacy significantly mediated the relationship between digital technostress and cyber moral disengagement. Specifically, the relationship of digital technostress on cyber moral disengagement operated through three mediation pathways: Ind1 [95% CI = (0.007, 0.063)], Ind2 [95% CI = (−0.238, −0.088)], and Ind3 [95% CI = (0.009, 0.040)], with none of the confidence intervals including zero, indicating that all mediation relationships were statistically significant. These results indicate that all three indirect paths exhibited statistically significant mediation relationships.

**Table 3:** Total, direct, and indirect effects with confidence intervals (*n* = 1980).

Indirect path	Effect	BootstrapSE	*95%CI*	
			LLCI	ULCI
Total effect	0.179	0.049	0.083	0.274
Direct effect	0.283	0.057	0.172	0.394
Indirect effect	−0.104	0.043	−0.191	−0.024
In1: Digital technostress→Self-efficacy→Cyber moral disengagement	0.032	0.015	0.007	0.063
In2: Digital technostress→Psychological resilience→Cyber moral disengagement	−0.158	0.039	−0.238	−0.088
In3: Digital technostress→Self-efficacy→Psychological resilience→Cyber moral disengagement	0.022	0.008	0.009	0.040

### Moderated multiple mediation analysis: the role of online self-control in the relationship between digital technostress and cyber moral disengagement

All variables were first standardized. Controlling for gender, grade level, only-child status, and daily internet usage duration, Model 15 of PROCESS macro version 4.1 was used to examine the moderating role of online self-control in the relationship of digital technostress on cyber moral disengagement via psychological resilience, with results presented in Table 4. The interaction between digital technostress and online self-control was significantly associated with cyber moral disengagement (*β* = 0.363, *SE* = 0.420, *t* = 3.102, *p* < 0.01), indicating that online self-control moderated this direct relationship. Additionally, the interaction between psychological resilience and online self-control was significantly related to cyber moral disengagement (*β* = −0.109, *SE* = 0.043, *t* = −2.504, *p* < 0.05), suggesting that online self-control also moderated the indirect relationship through psychological resilience.

**Table 4:** Moderated multiple mediation analysis.

Variable	Psychological resilience			Self-efficacy			Cyber moral disengagement		
	*β*	SE	*t*	*β*	SE	*t*	*β*	SE	*t*
Constant	−0.146	0.233	−0.627	0.231	0.260	0.887	0.897	0.218	4.115^***^
Gender	0.094	0.108	0.870	−0.081	0.121	−0.665	−0.158	0.102	−1.554
Grade	0.109	0.056	1.956	−0.025	0.063	−0.403	−0.137	0.052	−2.615^**^
Only child or not	−0.055	0.095	−0.578	−0.004	0.106	−0.037	−0.263	0.089	−2.947^**^
Daily internet usage time	−0.026	0.039	−0.662	−0.027	0.044	−0.615	−0.183	0.037	−0.496
Digital technostress	0.480	0.045	10.797^***^	−0.214	0.050	−4.307^***^	0.219	0.057	3.823^***^
Self-efficacy							−0.113	0.047	−2.419^*^
Psychological resilience							−0.374	0.055	−6.801^***^
Online self-control							0.363	0.047	7.684^***^
Digital technostress × Online self-control							0.130	0.420	3.102^**^
Self-efficacy × Online self-control							−0.056	0.043	−1.324
Psychological resilience × Online self-control							−0.109	0.043	−2.504^*^
*R^2^*	0.242			0.051			0.356		
*F*	24.844^***^			4.217^***^			19.304^***^		

*N* = 1980. *p<0.05; **p<0.01; ***p<0.001.

### These findings provide partial support for hypothesis 5

**Figure 2:**
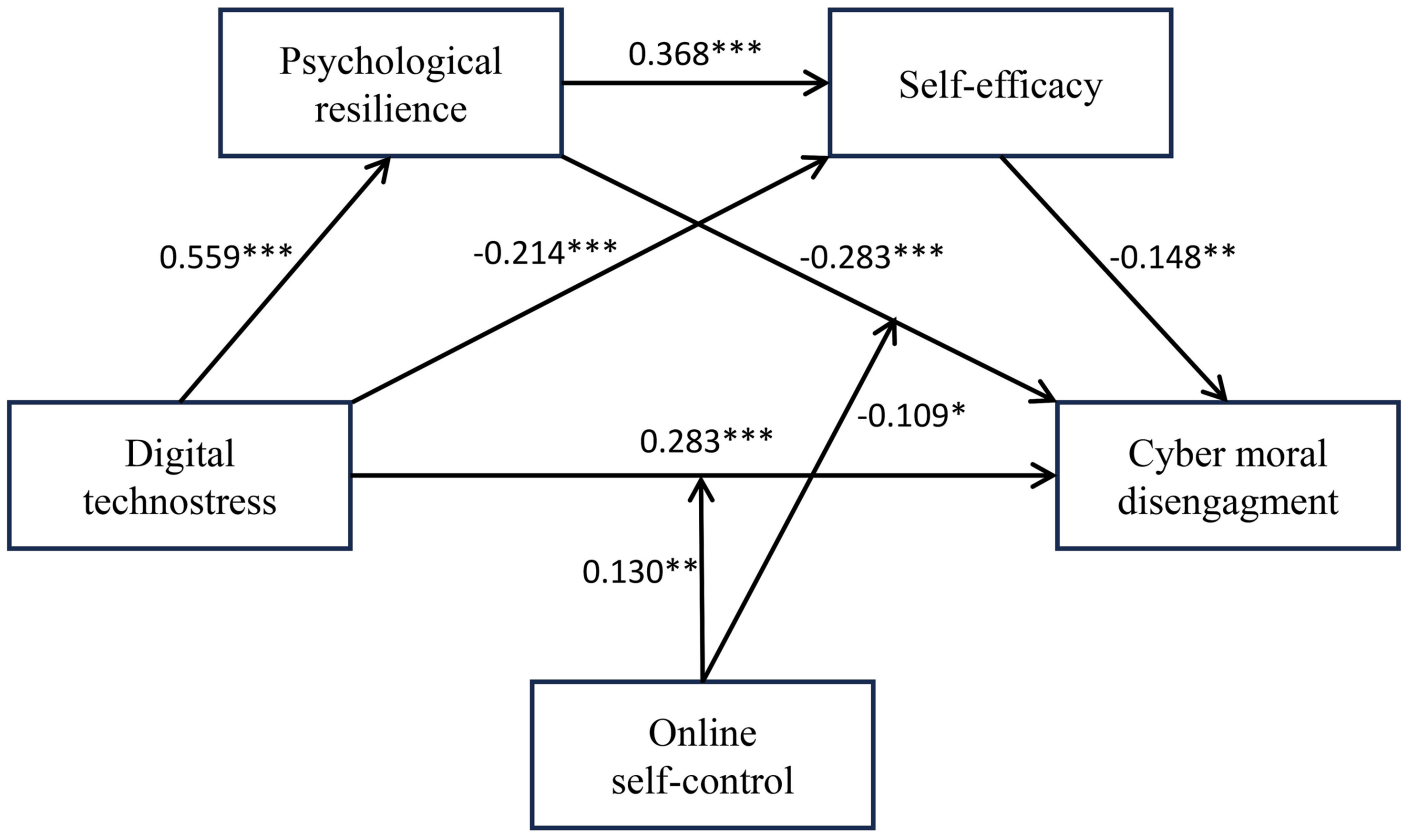
Moderated multiple mediation model.

In summary, the results suggest that digital technostress exerts an indirect relationship on cyber moral disengagement via the multiple mediating roles of psychological resilience and self-efficacy, with online self-control further moderating specific mediation pathways, as depicted in Figure 2. In adherence to contemporary reporting standards, we confirm that all statistically significant relationships are supported by accompanying effect sizes (*β*, *R^2^*, and indirect effects), demonstrating the practical relevance of our model beyond mere statistical significance. The model exhibits robust explanatory power, accounting for a large amount of variance in psychological resilience (*R^2^* = 0.370, *p* < 0.001) and a medium amount of variance in cyber moral disengagement (*R^2^* = 0.207, *p* < 0.001). Analysis of standardized path coefficients reveals that digital technostress exerts a dominant positive association with psychological resilience (*β* = 0.559, *p* < 0.001), indicating a large practical effect. Conversely, both psychological resilience (*β* = −0.283, *p* < 0.001) and self-efficacy (*β* = −0.148, *p* < 0.01) make significant medium-sized unique negative contributions to cyber moral disengagement. Critically, the strong suppressive effect of the indirect path through resilience [Effect = −0.158, *95% CI* (−0.238, −0.088)] represents a large negative indirect effect, underscoring its essential role as a protective factor. While the direct effect from technostress to disengagement remains significant (*β* = 0.283, *p* < 0.001), the significant interaction terms-such as digital technostress × online self-control (*β* = 0.130, *p* < 0.01) reveal small but practically meaningful moderating influences, suggesting that self-control significantly modulates the strength of these core associations. These effect size metrics collectively affirm that the statistically reliable findings possess substantial empirical and practical utility in understanding factors related to university students’ cyber-deviance.

**Figure 3:**
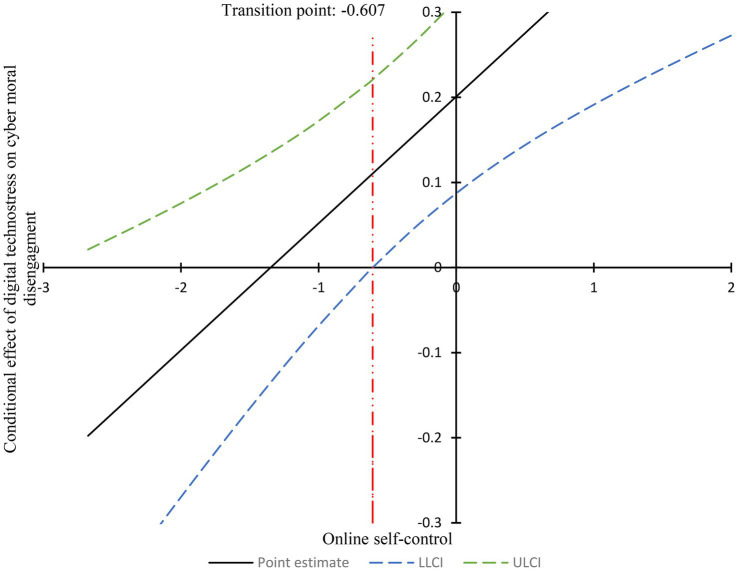
Conditional effect of digital technostress on cyber moral disengagement across levels of online self-control. LLCI, Lower level of confident interval; ULCI, Upper level of confident interval.

To further examine the moderating role of online self-control, the Johnson-Neyman (J-N) technique was applied to conduct simple slope analyses and identify the transition points at which moderation relationships became significant. Conditional relationships were observed, such that the relationship between digital technostress and cyber moral disengagement varied depending on whether participants’ online self-control scores were above or below the transition point. The J-N analysis revealed that when online self-control scores were below −0.607 (0.607 standard deviations below the mean of 2.020), digital technostress had a statistically significant relationship on cyber moral disengagement. As shown in Figure 3, when online self-control exceeded 2.020, the 95% confidence interval for the conditional relationship of digital technostress on cyber moral disengagement did not include zero, indicating a significant relationship. Conversely, when online self-control was below 2.020, the 95% confidence interval included zero, suggesting a non-significant relationship. These results provide evidence that online self-control moderates the relationship between digital technostress and cyber moral disengagement.

**Figure 4:**
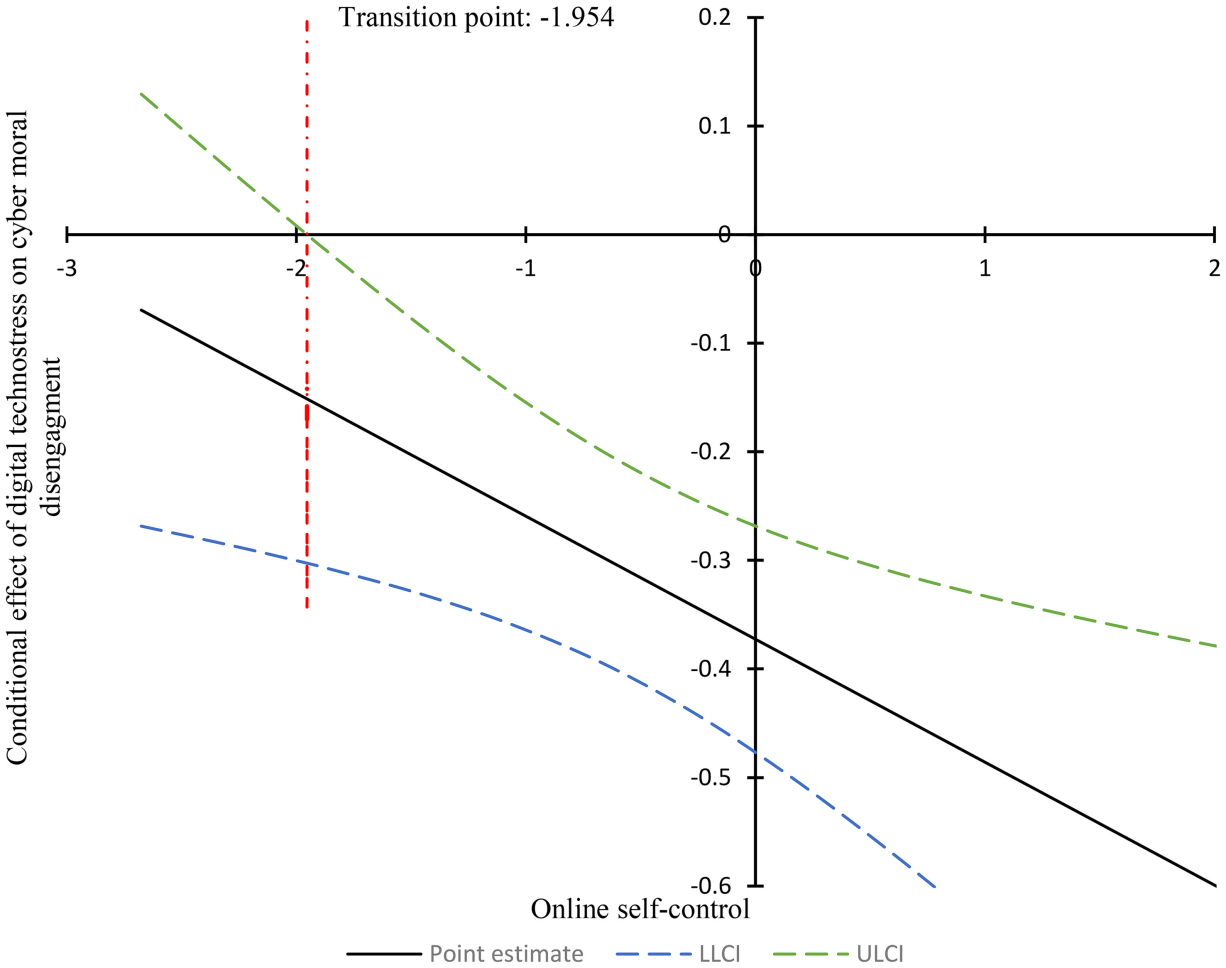
Conditional effect of psychological resilience on cyber moral disengagement across levels of online self-control. LLCI, Lower level of confident interval; ULCI, Upper level of confident interval.

The Johnson-Neyman analysis further identified a transition point for online self-control in the relationship between psychological resilience and cyber moral disengagement at −1.954, corresponding to 1.956 standard deviations below the mean of 1.356. As illustrated in Figure 4, when online self-control exceeded 1.356, psychological resilience was significantly associated with cyber moral disengagement, whereas scores below this threshold indicated a non-significant relationship. These findings suggest that online self-control acts as a protective factor, buffering the relationship between psychological resilience and cyber moral disengagement.

## Discussion

Although prior studies have documented a relationship between stress and behavioral intentions (Terp et al., 2019; Wu and Adamsk, 2021), the connection between digital technostress and cyber moral disengagement, along with the potential mediating and moderating mechanisms, remains underexplored. Our theoretical framework integrates the Theory of Planned Behavior (TPB) and Cognitive Load Theory (CLT) to provide a multi-level understanding of cyber moral disengagement. TPB serves as the overarching structure, framing ethical online conduct as a volitional behavior influenced by intentions and perceived behavioral control. However, TPB does not fully explain how external stressors disrupt this process. We introduce CLT to provide this critical explanatory mechanism. We posit that digital technostress imposes a high extraneous cognitive load, depleting the finite cognitive resources necessary for deliberate moral reasoning and self-regulation. This cognitive depletion makes individuals more susceptible to employing mental shortcuts, such as moral disengagement, thus weakening the link between ethical intentions and behavior. Drawing on the TPB (Ajzen, 1991) and CLT (Sweller, 1988), this study proposed a model in which psychological resilience and self-efficacy act as mediators, while online self-control operates as a moderator. The results indicated that psychological resilience and self-efficacy exerted both independent and sequential mediating relationships on the relationship between digital technostress and cyber moral disengagement. Furthermore, online self-control moderated the relationships of digital technostress and psychological resilience on cyber moral disengagement. The findings further suggested that the aforementioned relationships were significantly attenuated as online self-control increased.

First, consistent with Hypothesis 1, the results indicated a significant positive association between digital technostress and cyber moral disengagement. This finding indicates that in environments characterized by pervasive digital technology, prolonged exposure to information overload and technological complexity may induce cognitive fatigue (Cezar and Maçada, 2023) and emotional exhaustion, thereby diminishing individuals’ adherence to online behavioral norms. Under conditions of elevated stress, individuals may be more prone to neglect ethical responsibilities in online environments, thereby increasing the probability of engaging in cyber moral disengagement. Furthermore, this result suggests that technological stressors in the external environment may indirectly induce normative deviations in cyberspace by undermining individuals’ judgment and self-regulatory capacities (Peregrin, 2018).

Second, the findings support Hypothesis 2, demonstrating that psychological resilience mediates the relationship between digital technostress and cyber moral disengagement. Individuals exposed to digital technostress frequently encounter negative cognitive appraisals, which subsequently undermine their psychological resilience. Prior research has demonstrated that psychological resilience is linked to negative behavioral tendencies (Simmons et al., 2022) and that psychosocial and behavioral factors are related to online deviant behaviors (Brewer et al., 2023). Building on these findings, the present study extends the contextual focus by examining cyber moral disengagement specifically among college students, rather than concentrating solely on general digital deviance. Moreover, while previous studies have predominantly concentrated on psychological and educational domains, the present study introduces a novel empirical perspective on the psychological mechanisms underlying cyber moral disengagement.

Third, the results support Hypothesis 3, indicating that self-efficacy mediates the relationship between digital technostress and cyber moral disengagement, consistent with prior research (Luo and Bussey, 2019). Individuals exposed to digital technostress frequently question their ability to manage technological challenges, resulting in a reduced sense of control and increased perceptions of technological helplessness (Wang et al., 2025). When such adverse experiences persist, individuals may engage in prolonged self-reflection and self-denial, leading to diminished self-efficacy (Kim and Lee, 2021). Prior studies have shown that diminished self-efficacy undermines individuals’ internal regulatory mechanisms, increasing the likelihood of deviation from normative standards during moral judgment and thereby elevating the incidence of disengaged behaviors (Touloupis and Athanasiades, 2020). Within online environments, individuals with low self-efficacy frequently lack confidence in fulfilling their cyber moral responsibilities, demonstrate reduced willingness to adhere to online regulations, and consequently exhibit behavioral tendencies such as shirking responsibility and violating norms.

Fourth, the findings support Hypothesis 4, suggesting that the relationship between digital technostress and cyber moral disengagement is fully mediated through the sequential relationships of psychological resilience and self-efficacy. Individuals exposed to prolonged high levels of digital technostress are more likely to experience energy depletion and heightened cognitive load in online contexts, which can undermine psychological resilience. Psychological resilience fundamentally derives from the capacity to effectively mobilize internal and external resources to achieve positive adaptation when confronted with challenges and stressful situations (White et al., 2023). When this adaptive process is disrupted, individuals become more susceptible to emotional dysregulation (Weires et al., 2025) and constrained coping strategies, creating a self-perpetuating cycle of negative cognition and affect. Specifically, a decline in self-efficacy reduces individuals’ confidence in managing online conflicts and adhering to online norms, thereby eliciting avoidant behaviors (Odacı and Çelik, 2017) and a diminished sense of responsibility. This, in turn, further depletes psychological resilience, rendering individuals increasingly vulnerable under sustained online stress. Impaired self-efficacy not only directly diminishes self-regulatory capacity and self-restraint (Senécal et al., 2000) but also fosters greater reliance on short-term emotional release strategies, thereby heightening the likelihood of moral transgressions-such as cyber moral disengagement-in anonymous and low-accountability online environments. Therefore, it can be inferred that individuals experiencing high levels of digital technostress, when lacking sufficient psychological resilience and self-efficacy, are more prone to developing a stable pattern of cyber moral disengagement in online contexts.

Fifth, the results indicate that online self-control moderates the relationships of digital technostress and psychological resilience on cyber moral disengagement. Specifically, J-N analysis revealed that the transition points of online self-control significantly is associated with the relationships between digital technostress and cyber moral disengagement, as well as between psychological resilience and cyber moral disengagement. As individuals’ levels of online self-control increase, the relationships of digital technostress and psychological resilience on cyber moral disengagement become more pronounced. In other words, individuals with higher levels of online self-control are better able to regulate their attention and behavioral choices, thereby sustaining internal cognitive stability and moral self-discipline (Mensah et al., 2024). Within this context, digital technostress and psychological resilience act as critical predictors of cyber moral disengagement (Liu et al., 2022). Conversely, individuals with lower levels of online self-control are more vulnerable to external environmental relationships (Strömbäck et al., 2023), which may undermine their adherence to moral norms. Consequently, the relationships of digital technostress and psychological resilience on cyber moral disengagement are attenuated or non-significant among individuals with lower online self-control.

Sixth, the results do not support the hypothesis that online self-control moderates the relationship between self-efficacy and cyber moral disengagement. This outcome may reflect the possibility that self-efficacy activates moral self-awareness (Tangney et al., 2007), a mechanism widely recognized for fostering prosocial and ethical behavior. Even in cases of low online self-control, individuals with high self-efficacy may still rely on intrinsic moral awareness to regulate their online actions (Berte et al., 2021). While online self-control can mitigate the occurrence of cyber moral disengagement behaviors, the direct relationship of self-efficacy on online moral literacy appears to be more decisive than its potential moderating role (Taba et al., 2022). Furthermore, factors beyond moral self-awareness may also contribute to the complex interplay between online self-control and cyber moral disengagement. Future research should explore these additional factors to clarify the mechanisms underlying the relationships of online self-control in digital moral contexts.

This investigation offers significant insights into the interconnections linking digital technostress, psychological resilience, self-efficacy, online self-control, and cyber moral disengagement. Nevertheless, the results must be interpreted cautiously concerning their generalizability. The sampling frame, which consisted exclusively of Chinese university students, strongly suggests that cultural elements are likely to play a significant and nuanced role in shaping the observed associations. Within the Chinese cultural context, several factors could uniquely influence these findings. For instance, the manifestation or perceived intensity of digital technostress may differ significantly when compared to Western contexts (Giray et al., 2024). The demanding academic environment, alongside the pervasive integration of digital technology in daily life and strong societal expectations for both professional and academic success, can amplify students’ digital dependency and fear of missing out (FOMO). This, in turn, contributes distinct dimensions to the concept of technostress. Furthermore, the collectivist nature of Chinese society, which often emphasizes social conformity and group harmony, can affect how individuals cope with and experience stress, including digital stressors. The development and expression of psychological resources like resilience and self-efficacy are also subject to cultural modulation (Kim and Zalaquett, 2025). While these constructs possess universal validity, the specific coping mechanisms employed or the degree of personal empowerment felt by individuals may be shaped by cultural norms. These norms govern emotional expression, seeking support, and the balance between individual agency versus collective responsibility. For example, within a collectivist framework, self-efficacy might be tightly associated with social contributions or academic performance. Similarly, the practice of online self-control is likely influenced by the cultural premium placed on hierarchical obedience and self-discipline. The perceived social costs associated with online moral transgressions, often connected to “face” (mianzi) and reputation within tight-knit social networks, might establish a stronger external motivation for self-control, distinguishing it from purely individualistic motives. Most crucially, cyber moral disengagement itself may operate under unique cultural influences. The interpretation of “moral” conduct online, the justification mechanisms used, and the perceived severity of moral transgressions can all be informed by specific cultural values, traditional ethical principles, and the evolving regulatory landscape of the Chinese internet. Behaviors considered severely unethical in one culture, for example, might be rationalized differently in another, potentially altering the pathways through which technostress might lead to moral disengagement (Vitolla et al., 2021; Boski et al., 2025). Therefore, although these findings establish a robust model within the context of Chinese university students, their direct applicability to other cultural or social contexts, such as Western individualistic societies or different age demographics, is limited. Future comparative, cross-cultural studies are imperative to investigate the cultural-specific variations and the universality of these relationships. Such research would significantly enrich our theoretical understanding and facilitate the development of more broadly applicable interventions. Comparative work can help delineate which aspects of the established model are culturally robust and which are uniquely shaped by the Chinese socio-digital environment.

### Limitations

This study, while offering valuable insights into the complex relationships surrounding digital technostress and cyber moral disengagement, is subject to several methodological and contextual limitations that warrant careful consideration in future research. First, a primary methodological constraint stems from the cross-sectional nature of its design. Data collected at a single point in time fundamentally precludes the establishment of causal relationships among the examined variables, including digital technostress, psychological resilience, self-efficacy, online self-control, and cyber moral disengagement. While our findings reveal significant associations and intricate patterns within our proposed moderated mediation model, these results should therefore be interpreted as highlighting correlational links rather than definitive cause-and-effect pathways. Future investigations would greatly benefit from employing longitudinal designs to more robustly explore the dynamic interplay and temporal precedence of these constructs. For instance, collecting data across at least three waves at six-month intervals would provide a stronger empirical basis for inferring potential causal links. Second, the study’s exclusive reliance on self-reported questionnaires for all measures raises concerns about common method bias (CMB). Although procedural remedies were implemented to minimize this potential bias (e.g., ensuring anonymity and spatially separating variable measurements), and statistical diagnostics (Harman’s single-factor test and VIF values) indicated that CMB was not a severe issue in our data, these measures do not entirely rule out its presence. Harman’s single-factor test, in particular, has acknowledged limitations in definitively addressing CMB. Thus, while our statistical analyses suggest that CMB is unlikely to invalidate our primary conclusions, future research should integrate multiple data collection methods, such as behavioral observations, peer evaluations, or experimental manipulations, and employ more robust statistical techniques (e.g., marker variable approaches or CFA-based procedures) to further mitigate and rigorously assess potential common method variance. Finally, the cultural specificity of our sample presents a significant contextual limitation. As our participants were exclusively Chinese university students, the direct generalizability of these findings to other cultural or social contexts is constrained. Cultural factors, including distinct social norms, educational systems, digital infrastructure, and traditional ethical frameworks, may uniquely influence the experience of digital technostress, the development and expression of psychological resources, the practice of online self-control, and the manifestation of cyber moral disengagement. While our study contributes a robust model within this specific context, future work should prioritize cross-cultural replication and comparative analyses to explore the universality and culturally specific variations of these relationships, thereby strengthening the external validity and applicability of the proposed model across diverse populations.

There are considerations regarding measurement validity that warrant discussion. While most of our measures demonstrated good psychometric properties, the confirmatory factor analysis for the Online Self-Control scale yielded a borderline fit on one index (TLI = 0.897). Although other key fit indices (e.g., CFI = 0.920, RMSEA = 0.066, SRMR = 0.020) and the internal consistency (Cronbach’s *α* = 0.884, McDonald’s *ω* = 0.917) were acceptable, the suboptimal TLI value suggests that the model’s fit is adequate rather than excellent. While we believe this measure was sufficiently robust for testing the relationships in our proposed model, we recommend that future research using this scale should aim to further validate its factor structure, perhaps in different populations or contexts, to enhance its psychometric robustness.

### Implications

The findings of this study have important practical implications for designing interventions aimed at reducing cyber moral disengagement among college students. Specifically, intervention strategies should primarily target situational risk factors such as information overload, social pressure, and attentional distraction that are closely associated with digital technostress. Furthermore, the study underscores the mediating roles of psychological resilience and self-efficacy in influencing cyber moral disengagement. Psychological resilience can be strengthened through cognitive behavioral training, while self-efficacy can be enhanced via goal setting, role modeling, and positive feedback. Prior research indicates that goal-setting interventions effectively improve behavioral motivation and achievement expectations, reinforce self-control, and facilitate moral decision-making in complex online environments, thereby promoting overall online moral literacy.

The moderating relationship of online self-control highlights the importance of fostering self-regulatory behaviors to mitigate cyber moral disengagement. Universities should consider integrating online self-control training into curricula to develop students’ abilities in delayed gratification, behavioral self-discipline, and emotional regulation, enhancing their capacity to resist online temptations and make ethical judgments. Practical measures may include thematic lectures, situational simulations, structured debates, and regularized evaluation mechanisms to monitor the effectiveness of interventions and prevent uncontrolled Internet use. Online self-control scores can be used to identify critical thresholds: students scoring below these thresholds may require closer supervision and individualized guidance, while those above may benefit from encouragement and positive reinforcement to consolidate good habits. At the individual level, students should be encouraged to engage in self-monitoring, strengthen reflective practices, actively resist undesirable online temptations, and cultivate an ethical online persona. Collectively, these recommendations provide a framework for multi-level, structured interventions, laying a solid foundation for fostering a cohort of college students with strong online behavioral competencies in the digital era.

## Conclusion

In summary, this study contributes to the literature on online behavioral processes by exploring the intricate patterns linking digital technostress and cyber moral disengagement among university students. Our findings reveal that psychological resilience and self-efficacy are associated with both independent and sequential mediating roles in the relationship between digital technostress and cyber moral disengagement. Furthermore, online self-control is identified as a significant moderator in the associations of digital technostress and psychological resilience with disengaged behavior. Overall, this research provides a novel perspective on the psychological correlates and relational dynamics underlying digital technostress, and offers valuable implications for understanding and potentially addressing cyber moral disengagement among college students. Such implications suggest the relevance of targeted training interventions, strategies to strengthen individual psychological resources and self-regulatory capacities, and a nuanced understanding of the complex interplay between internal psychological factors and the external digital environment in the context of these observed relationships.

## Data Availability

The original contributions presented in the study are included in the article/Supplementary material, further inquiries can be directed to the corresponding author/s.
