# Supplementary material for: The relationship between digital technostress and cyber moral disengagement among college students: a moderated mediation model of psychological resilience, self-efficacy, and online self-control
Source: Front Psychol. 2025 Nov 11;16:1706794. doi: 10.3389/fpsyg.2025.1706794 (PMC12645413; doi:10.3389/fpsyg.2025.1706794)
Supplement: Supplementary file 1 [file Supplementary_file_1.docx]

Supplementary Material

Research Questionnaire

Dear Student,

Thank you for participating in this survey. This questionnaire focuses on university students' digital technostress, cyber moral disengagement, online self-control, psychological resilience, and self-efficacy. There are no right or wrong answers. Please respond based on your actual experiences and situations. All data will be kept strictly confidential and used solely for scientific research purposes. The survey will take approximately 30 minutes to complete. Your thoughtful responses are highly valuable to our study. We greatly appreciate your time and cooperation.

**Section I: Demographic Profile​**

(1) ​Gender Identity:

　　A. Male　　　　　B. Female

(2) Academic Year: ​

　　A. First-year undergraduate　　　B. Second-year undergraduate

　　C. Third-year undergraduate　　 D. Fourth-year undergraduate

(3) Disciplinary Category:

　　A. Humanities　　　　　B. Natural Sciences　　　C. Engineering

　　D. Medical Sciences　　 E. Arts　　　　　　　　 F. Physical Education

(4) Only child status:

　　A. Yes B. No

(5) Place of origin:

　　A. Urban B. Rural

(6) Daily Internet usage duration (including both mobile and computer use):

　　A. Less than 1 hour B. 1–2 hours C. 3–5 hours

　　D. 6–8 hours E. More than 8 hours

**Section II:** **Digital technostress**

| Title | 1.Strongly Disagree | 2.Disagree | 3. Neutral | 4. Agree | 5.Strongly Agree |
| --- | --- | --- | --- | --- | --- |
| 1. Using digital technology enables me to complete tasks more quickly. |  |  |  |  |  |
| 2.Using digital technology improves the quality of my learning. |  |  |  |  |  |
| 3.Using digital technology makes my learning easier. |  |  |  |  |  |
| 4.Using digital technology enhances my efficiency in learning. |  |  |  |  |  |
| 5.I feel that the features of digital technology itself change frequently. |  |  |  |  |  |
| 6.I feel that my ability to use digital technology changes frequently. |  |  |  |  |  |
| 7.I feel that the ways of using digital technology change frequently. |  |  |  |  |  |
| 8.Digital technology forces me to work faster. |  |  |  |  |  |
| 9.Digital technology forces me to handle a workload beyond my capacity. |  |  |  |  |  |
| 10.Digital technology forces me to work under very tight schedules. |  |  |  |  |  |
| 11.I am compelled to change my work habits to adapt to new technologies. |  |  |  |  |  |
| 12.My academic burden has increased due to the growing complexity of technology. |  |  |  |  |  |
| 13.Digital technology has reduced the time I spend with my family. |  |  |  |  |  |
| 14.Even during vacations, I must stay connected with teachers and classmates. |  |  |  |  |  |
| 15.I have to sacrifice holidays and weekends to keep up with new technologies. |  |  |  |  |  |
| 16.I feel that my personal academic life is invaded by digital technology. |  |  |  |  |  |
| 17.My lack of understanding of digital technology prevents it from satisfyingly handling my tasks. |  |  |  |  |  |
| 18.It takes me a long time to understand and use new technologies. |  |  |  |  |  |
| 19.I do not have enough time to study and improve my technical skills. |  |  |  |  |  |
| 20.I find that my peers know more about digital technology than I do. |  |  |  |  |  |
| 21.I often find new technologies too complex to understand and use. |  |  |  |  |  |
| 22.I feel that my academic performance is constantly threatened due to new technologies. |  |  |  |  |  |
| 23.To avoid underperforming, I must continuously update my skills. |  |  |  |  |  |
| 24.I worry that my classmates possess more updated technological skills. |  |  |  |  |  |
| 25.Due to fear of underperforming, I do not share my knowledge with classmates. |  |  |  |  |  |
| 26.Due to fear of underperforming, I feel that knowledge sharing among classmates has reduced. |  |  |  |  |  |
| 27.In our field of study, technological advancements are constantly evolving. |  |  |  |  |  |
| 28.In our field of study, digital technology software changes frequently. |  |  |  |  |  |
| 29.In our field of study, digital technology hardware changes frequently. |  |  |  |  |  |
| 30.In our field of study, digital technology networks are frequently upgraded. |  |  |  |  |  |
| 31.I would use digital technology to complete my academic tasks if I see others using it first. |  |  |  |  |  |
| 32.I would use digital technology to complete my academic tasks if someone is available to show or tell me how to operate it. |  |  |  |  |  |
| 33.I would use digital technology to complete my academic tasks if I have sufficient time for the tasks required by the mobile application. |  |  |  |  |  |
| 34.I would use digital technology to complete my academic tasks if built-in help functions are available for assistance. |  |  |  |  |  |
| 35.I would use digital technology to complete my academic tasks if I have previously used similar applications for the same work. |  |  |  |  |  |
| 36.Activities requiring me to use digital technology make me feel exhausted. |  |  |  |  |  |
| 37.My digital technology activities make me feel tired. |  |  |  |  |  |
| 38.Using digital technology all day is stressful for me. |  |  |  |  |  |
| 39.My digital technology activities leave me feeling drained. |  |  |  |  |  |
| 40.I often feel fatigued when using digital technology. |  |  |  |  |  |
| 41.I often feel physically exhausted when using digital technology. |  |  |  |  |  |
| 42.I often feel emotionally exhausted when using digital technology. |  |  |  |  |  |
| 43.I often think, "I can't take it anymore," when using digital technology. |  |  |  |  |  |
| 44.Digital technology helps improve the quality of my learning. |  |  |  |  |  |
| 45.Digital technology helps enhance my academic productivity. |  |  |  |  |  |
| 46.Digital technology enables me to accomplish more academic work that would otherwise be impossible. |  |  |  |  |  |
| 47.Digital technology helps me perform my academic tasks better. |  |  |  |  |  |

**Section III:** **Cyber moral disengagement**

The following statements describe various behaviors related to internet use. Please read each item carefully and select the option that best corresponds to your actual situation based on your personal experience.

| Title | 1.Strongly Disagree | 2.Disagree | 3. Neutral | 4. Agree | 5.Strongly Agree |
| --- | --- | --- | --- | --- | --- |
| 1. Excessive online engagement in making friends with strangers. |  |  |  |  |  |
| 2.Sharing phone numbers or addresses with unfamiliar online acquaintances. |  |  |  |  |  |
| 3.Disclosing personal privacy to unfamiliar online acquaintances. |  |  |  |  |  |
| 4.Frequently making phone calls with online acquaintances. |  |  |  |  |  |
| 5.Verbally attacking others online. |  |  |  |  |  |
| 6.Posting offensive symbols or images online. |  |  |  |  |  |
| 7.Leaving insulting remarks in others' blogs or microblog (Weibo) comment sections. |  |  |  |  |  |
| 8.Content opposing core societal values/leadership |  |  |  |  |  |
| 9.Posting false information about others online. |  |  |  |  |  |
| 10.Browsing violent images, text, or videos online. |  |  |  |  |  |
| 11.Browsing pornographic websites. |  |  |  |  |  |
| 12.Searching for information related to "sex" online. |  |  |  |  |  |
| 13.Viewing or downloading pornographic videos or images online. |  |  |  |  |  |
| 14.Posting harassing advertisements on QQ, Weibo, or via email. |  |  |  |  |  |
| 15.Participating in human flesh searches (doxing / online manhunts). |  |  |  |  |  |
| 16.Uploading others' private photos or videos without permission. |  |  |  |  |  |
| 17.Posting or spreading false information/rumors online. |  |  |  |  |  |
| 18.Selling products online that do not match their physical description. |  |  |  |  |  |
| 19.Frequently purchasing lottery tickets online. |  |  |  |  |  |

**Section IV: Online self-control**

The following section pertains to Internet usage behaviors. Please select the options that best reflect your actual situation.

| Title | 1.Strongly Disagree | 2.Disagree | 3. Neutral | 4. Agree | 5.Strongly Agree |
| --- | --- | --- | --- | --- | --- |
| 1.I plan what I need to do before going online. |  |  |  |  |  |
| 2.I often miss meals or sleep because I'm online. |  |  |  |  |  |
| 3.Being online often disrupts my daily routine. |  |  |  |  |  |
| 4.I often have a bad temper online. |  |  |  |  |  |
| 5.I often browse the internet aimlessly and on a whim. |  |  |  |  |  |
| 6.I often actively control my online time. |  |  |  |  |  |
| 7.I easily get into arguments or conflicts with others online. |  |  |  |  |  |
| 8.The internet has many negative effects on my life. |  |  |  |  |  |
| 9.My emotions change frequently and unpredictably online. |  |  |  |  |  |
| 10.I can effectively balance online entertainment, study, and work. |  |  |  |  |  |
| 11.I know how to prevent internet use from interfering with my life. |  |  |  |  |  |
| 12.I prefer skill-based online games over intelligence-based ones. |  |  |  |  |  |
| 13.I often pursue novelty and excitement without regard for online safety. |  |  |  |  |  |
| 14.I am very capable of changing uncivilized online behaviors. |  |  |  |  |  |
| 15.I am cautious online. |  |  |  |  |  |
| 16.I enjoy seeking novelty and excitement online. |  |  |  |  |  |
| 17.Online, I sometimes do things I know I shouldn't. |  |  |  |  |  |
| 18.My online behavior often feels out of my control. |  |  |  |  |  |
| 19.I believe I can utilize online resources effectively. |  |  |  |  |  |
| 20.I often indulge myself online. |  |  |  |  |  |
| 21.I often act impulsively online. |  |  |  |  |  |
| 22.My sense of time diminishes after being online. |  |  |  |  |  |
| 23.When online, I become oblivious to everything around me. |  |  |  |  |  |
| 24.I get annoyed if someone interrupts me while I'm online. |  |  |  |  |  |
| 25.When studying or working online, I complete planned tasks despite difficulties or distractions. |  |  |  |  |  |
| 26.I have strong self-control regarding my internet use. |  |  |  |  |  |
| 27.I can quickly calm down after the excitement of online activities (e.g., chatting, gaming). |  |  |  |  |  |
| 28.My online persona feels like a different person from my real-life self. |  |  |  |  |  |
| 29.After being online for a while, I often feel somewhat disoriented when switching to other activities. |  |  |  |  |  |
| 30.I often think "just one more minute" but end up online much longer. |  |  |  |  |  |
| 31.I lack patience for complex or difficult tasks online and struggle to continue. |  |  |  |  |  |
| 32.I know what is and isn't appropriate to do online. |  |  |  |  |  |
| 33.When online, I often forget about hunger or fatigue. |  |  |  |  |  |
| 34.When I need to study or work, I can stop using the internet promptly. |  |  |  |  |  |

**Section V:** **Psychological resilience**

The following statements reflect various aspects of an individual's psychological resilience. Please compare each statement with your actual situation and select the description that best applies to you.

| Title | 1.Strongly Disagree | 2.Disagree | 3. Neutral | 4. Agree | 5.Strongly Agree |
| --- | --- | --- | --- | --- | --- |
| 1.Overall, I am quite satisfied with myself.  My parents often show concern about my life at school in various ways. |  |  |  |  |  |
| 2.Minor issues in daily life easily affect my mood. |  |  |  |  |  |
| 3 I frequently keep in touch with my family. |  |  |  |  |  |
| 4.I am able to find friends to share joys and sorrows with. |  |  |  |  |  |
| 5.I am confident in my abilities. |  |  |  |  |  |
| 6.I often receive emotional support from my family. |  |  |  |  |  |
| 7.When facing difficulties, I usually make plans and follow them. |  |  |  |  |  |
| 8.When I feel upset or troubled, I can seek emotional comfort from my family. |  |  |  |  |  |
| 9.I think I am doing pretty well. |  |  |  |  |  |
| 10.I often feel down for no apparent reason. |  |  |  |  |  |
| 11.When I need help, I can largely rely on my friends. |  |  |  |  |  |
| 12.I always appear very confident to others. |  |  |  |  |  |
| 13.I am easily angered. |  |  |  |  |  |
| 14.When encountering difficulties, I often seek help from friends. |  |  |  |  |  |
| 15.Considering my strengths and weaknesses, I am relatively satisfied with myself. |  |  |  |  |  |
| 16.I am often overly sentimental. |  |  |  |  |  |
| 17.My parents often show concern about my life at school in various ways. |  |  |  |  |  |
| 18.I have at least one close friend with whom I can share everything. |  |  |  |  |  |
| 19.If I put in the effort, I can do things as well as or even better than others. |  |  |  |  |  |
| 20.Unpleasant events often bother me for a long time. |  |  |  |  |  |
| 21.There are people in my life (friends or teachers) who genuinely care about me. |  |  |  |  |  |
| 22.Often, I feel less capable than many people around me. |  |  |  |  |  |
| 23.My emotions are relatively stable. |  |  |  |  |  |
| 24.When I am confused, my friends (or teachers) often provide helpful advice. |  |  |  |  |  |
| 25.I usually prioritize and address issues based on their urgency and importance. |  |  |  |  |  |
| 26.When I am in difficulty, I often receive substantial support from my family. |  |  |  |  |  |
| 27.I do not like discussing my difficulties with friends. |  |  |  |  |  |
| 28.I am good at managing time effectively to solve problems. |  |  |  |  |  |
| 29.I still maintain close relationships with some of my old friends. |  |  |  |  |  |
| 30.My emotions fluctuate frequently. |  |  |  |  |  |
| 31. I often take proactive actions and strategies to solve problems and resolve conflicts. |  |  |  |  |  |

**Section VI:** **Self-efficacy**

The following questions relate to self-efficacy. Please select the option that best reflects your personal experience.

| Title | 1.Not at all true | 2.Hardly true | 3.Moderately true | 4.Exactly true |
| --- | --- | --- | --- | --- |
| 1.I can always manage to solve difficult problems if I try hard enough. |  |  |  |  |
| 2.I am confident that I could deal efficiently with unexpected events. |  |  |  |  |
| 3.I can remain calm when facing difficulties because I can rely on my problem-solving abilities. |  |  |  |  |
| 4.When I am confronted with a problem, I can usually find several solutions. |  |  |  |  |
| 5.Whatever happens, I am able to handle it. |  |  |  |  |
| 6.If someone opposes me, I can still find ways to achieve what I want. |  |  |  |  |
| 7.It is easy for me to stick to my goals and accomplish them. |  |  |  |  |
| 8.I am confident in my ability to respond effectively to sudden challenges. |  |  |  |  |
| 9.Thanks to my resourcefulness, I know how to handle unforeseen situations. |  |  |  |  |
| 10.If I put in enough effort, I can overcome most problems. |  |  |  |  |
